# All-2D ReS_2_ transistors with split gates for logic circuitry

**DOI:** 10.1038/s41598-019-46730-7

**Published:** 2019-07-17

**Authors:** Junyoung Kwon, Yongjun Shin, Hyeokjae Kwon, Jae Yoon Lee, Hyunik Park, Kenji Watanabe, Takashi Taniguchi, Jihyun Kim, Chul-Ho Lee, Seongil Im, Gwan-Hyoung Lee

**Affiliations:** 1 Department of Materials Science and Engineering, Yonsei University, Seoul, 03722 Korea; 2 Department of Materials Science and Engineering, Seoul National University, Seoul, 08826 Korea; 3 vdWMRC, Department of Physics, Yonsei University, Seoul, 03722 Korea; 4 KU‐KIST Graduate School of Converging Science and Technology, Korea University, Seoul, 02841 Korea; 5 Department of Chemical and Biological Engineering, Korea University, Seoul, 02841 Korea; 6 National Institute for Materials Science, Ibaraki, 305-0044 Japan

**Keywords:** Electronic devices, Electrical and electronic engineering, Electronic properties and devices

## Abstract

Two-dimensional (2D) semiconductors, such as transition metal dichalcogenides (TMDs) and black phosphorus, are the most promising channel materials for future electronics because of their unique electrical properties. Even though a number of 2D-materials-based logic devices have been demonstrated to date, most of them are a combination of more than two unit devices. If logic devices can be realized in a single channel, it would be advantageous for higher integration and functionality. In this study we report high-performance van der Waals heterostructure (vdW) ReS_2_ transistors with graphene electrodes on atomically flat hBN, and demonstrate a NAND gate comprising a single ReS_2_ transistor with split gates. Highly sensitive electrostatic doping of ReS_2_ enables fabrication of gate-tunable NAND logic gates, which cannot be achieved in bulk semiconductor materials because of the absence of gate tunability. The vdW heterostructure NAND gate comprising a single transistor paves a novel way to realize “all-2D” circuitry for flexible and transparent electronic applications.

## Introduction

Ultrathin two-dimensional (2D) semiconducting materials are useful for a number of electronic applications because of their unique properties originating from their atomically thin nature^1–5^. Among them, transition metal dichalcogenides (TMDs) have been actively studied as channel materials because of absence of short-channel effect^6,7^. It has been anticipated that a scale-down limit can be overcome by 2D semiconductors for higher integration of electronic devices. In this regard, “all-2D” devices comprising only 2D materials, *i.e*., van der Waals heterostructure (vdW) devices, have been demonstrated^8–11^. As emerging 2D materials, rhenium-based TMDs, such as ReS_2_ or ReSe_2_, have exhibited promising properties. Typically, few-layer TMDs outperform monolayer in terms of carrier mobility, while the band structure of TMDs transforms from direct to indirect band structure as the thickness changes from monolayer to few-layers, which limits their use for optoelectronic applications^12^. However, ReS_2_ and ReSe_2_ exhibit direct band structure at all thickness because of the distorted 1 T’ structure and weak interlayer coupling^13^. Despite the unique properties of ReS_2_ and ReSe_2_, there have been only limited studies on them, which reported preliminary results of transistors and photodetectors based on ReS_2_ and ReSe_2_ with conventional device geometry^14–19^.

To improve the device performance of 2D semiconductors, contact resistance and carrier scattering should be reduced. Significant contact resistance between deposited metal and 2D semiconducting channels severely deteriorates device performance due to the Fermi level pinning at the interface^20^, which makes it difficult to utilize the 2D semiconductors for practical applications required by the industry. In addition, because the band gap of TMDs increases with decreasing thickness, the Schottky barrier height for monolayer TMDs is significantly higher than that for few-layer TMDs, leading to a lower field-effect mobility^21,22^. The use of graphene electrodes has been considered as a solution because of the de-pinning of the graphene Fermi level and absence of chemical bonds at a stacked heterointerface of 2D layers^23^. In addition, it has been shown that hexagonal boron nitride (hBN), used as an ultraflat dielectric, suppresses charged impurity scattering, resulting in enhanced carrier mobility of hBN-encapsulated 2D materials^9,11,24^.

A number of logic devices based on 2D materials have been demonstrated by combinations of two different 2D transistors: n-type and p-type^5,25–28^. Even though the NMOS inverters using ReS_2_ have been demonstrated^19,27^, all of the logic devices reported so far are made of double devices (two connected devices). Therefore, for higher integration and functionality, a novel device geometry is required for logic devices. Because atomically thin 2D materials are highly sensitive to electric fields with no screening effect, it is possible to locally tune the densities and types of carriers, leading to electrically controlled p-n junction devices comprising graphene or WSe_2_^29–33^. Therefore, by locally tuning the Fermi level of a 2D channel, a multi-functional logic device can be realized in one 2D channel.

Here we report a NAND gate based on a single ReS_2_ channel with a graphene split gate. We first demonstrate ReS_2_ field-effect transistors (FETs) on an hBN substrate with graphene electrodes. The field-effect mobility (μ_FE_) of an n-type ReS_2_ FET is 35 cm^2^/V·s and the on/off ratio is as high as 10^6^ at room temperature. When the graphene split gate is used to locally modulate the channel, two regions, separately tuned by the split gates, act as independent transistors, which can be used as a gate-tunable NAND logic gate.

## Results

**Figure 1:**
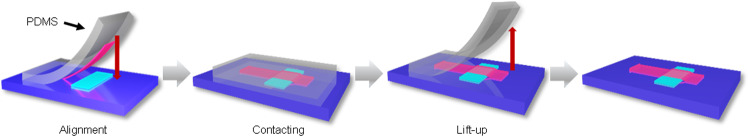
PDMS stacking process for fabrication of vdW heterostructure. The flake exfoliated directly on PDMS is aligned with a target flake on the SiO_2_ substrate. The PDMS stamp is smoothly lowered for conformal contact onto the bottom target flake on the substrate. The lift-up process follows after five minutes.

**Figure 2:**
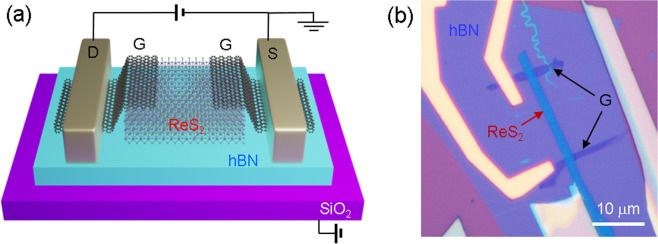
ReS_2_ FET with graphene contacts and hBN dielectric. (**a**) Schematic of ReS_2_ FET fabricated by stacking process. (**b**) Optical microscope image of fabricated tri-layer ReS_2_ FET. Because of anisotropic transport of ReS_2_, the elongated ReS_2_, which is longer along the *a*-axis, is selected as a channel.

For “all-2D” devices, a vdW heterostructure of ReS_2_, graphene, and hBN was fabricated by stacking the exfoliated nanosheets, as shown in Fig. 1. The 2D nanosheets were exfoliated directly onto a polydimethylsiloxane (PDMS) stamp and transferred onto another flake on the SiO_2_ substrate^22^. The ReS_2_ channel was placed on the hBN to reduce scattering from charged impurities of the substrate and interfacial impurities. As ReS_2_ has a smaller effective mass along the a-axis, it has anisotropy in conductance, leading to higher carrier mobility along the a-axis^27^. Therefore, the ReS_2_ flake, which has an elongated shape along the a-axis, was chosen as a channel. Graphene was used as an electrode to lower the Schottky barrier. Two pieces of graphene were placed on the ends of the ReS_2_ channel to form source and drain electrodes. After stacking, e-beam lithography and metal deposition (1 nm Cr/50 nm Au) were used to define the metal electrodes. Figure 2(a,b) show the schematic and optical microscopic (OM) images of the fabricated device.

**Figure 3:**
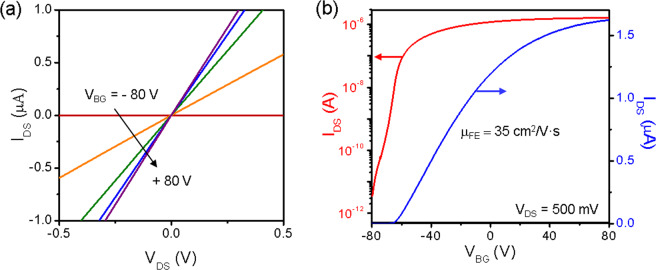
Electrical characteristics of tri-layer ReS_2_ FET. (**a**) Output curves (*I*_*DS*_ − *V*_*DS*_) of device. Linearity of output curves at all gate voltages indicates that graphene-ReS_2_ junction is Ohmic contact. (**b**) Transfer curves (*I*_*DS*_ − *V*_*BG*_) plotted in semi-log scale (red) and linear scale (blue). The ReS_2_ FET exhibits high field effect mobility of 35 cm^2^/V·s and on-off ratio of 10^6^.

The electrical performance of the tri-layer ReS_2_ FET on the hBN substrate with graphene electrodes is shown in Fig. 3. The few-layer graphene (>10 L) and thick hBN of 6.4 nm were used in the device. The output curves (*I*_*DS*_ − *V*_*DS*_) at different back gate voltages (*V*_*BG*_) clearly exhibit a gate-dependent linearity, as shown in Fig. 3(a). The linear output curves at small gate voltages indicate that the ReS_2_-graphene interface forms Ohmic contact even at low carrier concentrations. As shown in the transfer curves (*I*_*DS*_ − *V*_*BG*_) in Fig. 3(b), ReS_2_ exhibits n-type transport as typically observed in MoS_2_ or WS_2_^34^. Because of the low contact resistance and reduced carrier scattering in the vdW heterostructure device, higher mobility (μ_FE_ = 35 cm^2^/V·s) and a high on-off ratio (~10^6^) were extracted, compared with the same ReS_2_ FET with metal electrodes on a SiO_2_/Si substrate (Fig. S1). It should be noted that the field-effect mobility of a vdW heterostructure device of ReS_2_ is significantly greater than previously reported values (<10 cm^2^/V·s) of ReS_2_ devices with metal or graphene electrodes and SiO_2_ dielectric^14–19^. We also fabricated ReSe_2_ devices using the same procedure and device structure. As ReSe_2_ exhibits a smaller mobility of 6.6 cm^2^/V·s than ReS_2_, ReS_2_ was used for the logic device. (Fig. S2)

**Figure 4:**
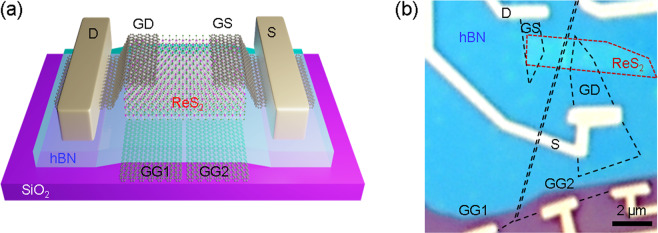
Tri-layer ReS_2_ FET with graphene split gates. (**a**) Schematic of device. GD, GS, GG1, and GG2 indicate graphene drain, graphene source, graphene gates 1 and 2, respectively. (**b**) Optical microscope image of fabricated device.

We modified the previous device structure to demonstrate the logic devices comprising a single 2D channel. To locally tune the ReS_2_ channel, graphene was inserted between the bottom hBN and SiO_2_ and patterned into split gates separated by a nanogap, as shown in Fig. 4(a). (See Fig. S3 for detail of the fabrication.) After exfoliation of the graphene on the SiO_2_ substrate, e-beam lithography is performed to define a nanogap on the graphene, followed by reactive-ion etching (RIE) etching for cutting. The patterned graphene split gates are annealed at 350 °C for 4 hrs in forming gas (5% H_2_ in Ar) to remove residue. After cleaning, other 2D flakes of hBN dielectric, ReS_2_ channel, and graphene electrodes were sequentially transferred by the PDMS stamping technique as described in Fig. 1. After stacking, e-beam lithography and metal deposition were conducted to form metal contacts to the source, drain, and gate graphene electrodes. The OM image in Fig. 4(b) shows the fabricated tri-layer ReS_2_ device with graphene split gates.

**Figure 5:**
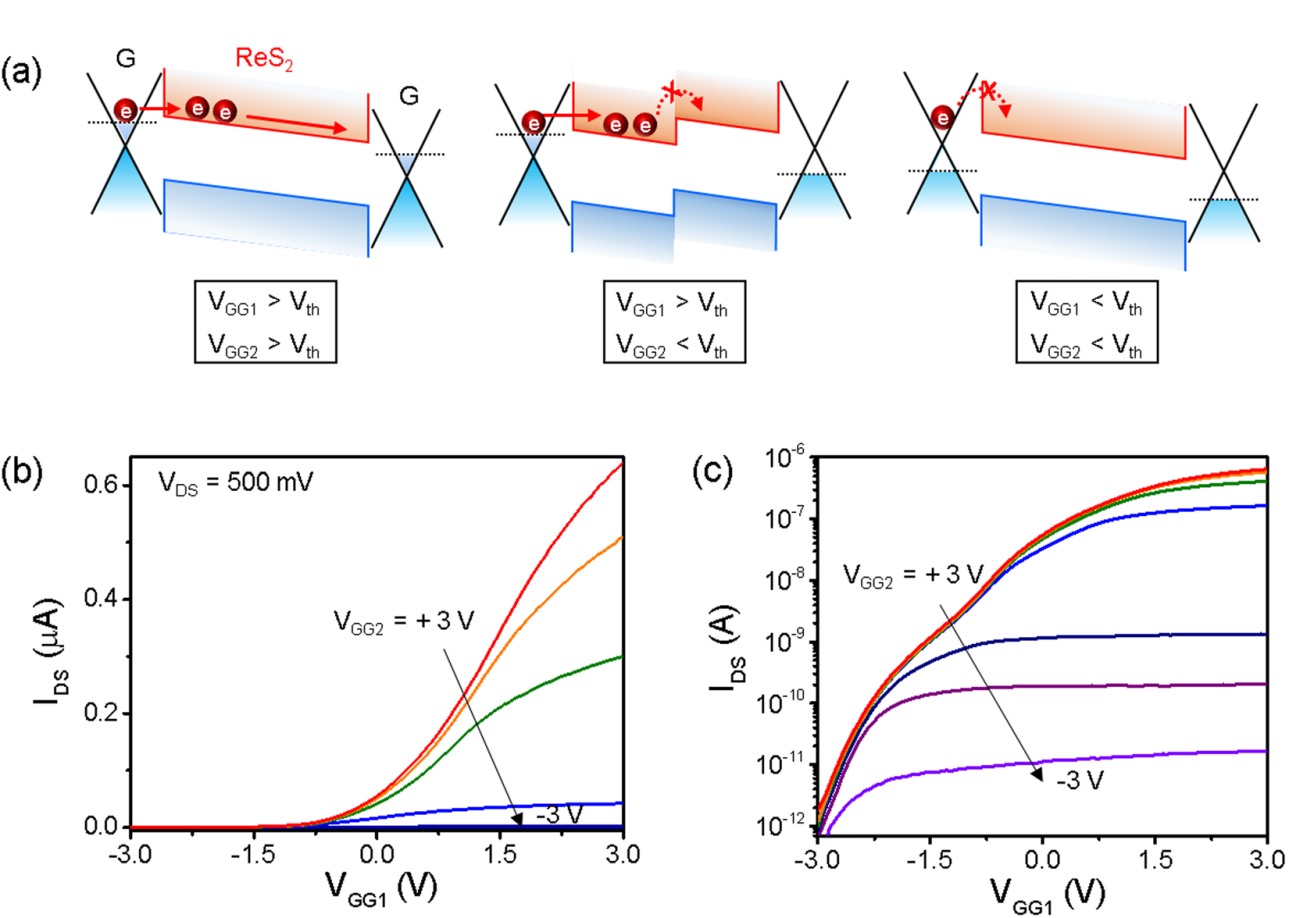
Band diagram and electrical modulation characteristics of the device. (**a**) Band alignment under various combinations of different gate voltages by split gates. When both gates are on (*V*_*GG1*_ and *V*_*GG1*_ > *V*_*th*_), charge carriers transport through ReS_2_ channel (left). If one of split gates is off (*V*_*GG1*_ > *V*_*th*_ and *V*_*GG1*_ < *V*_*th*_), half of channel is depleted and charge carriers do not transport (middle). When both gates are off (*V*_*GG1*_ and *V*_*GG1*_ < *V*_*th*_), charge carriers cannot be injected from graphene and transport through channel (right). (**b**,**c**) Transfer curves (*I*_*DS*_ − *V*_*GG1*_) of the ReS_2_ FET at varying *V*_*GG2*_ and fixed *V*_*GG1*_ in linear scale (**b**) and semi-log scale (**c**).

When different voltages are applied separately to the two graphene split gates (*V*_*GG1*_ and *V*_*GG2*_), the corresponding band alignments are shown in Fig. 5(a). When both gate voltages of *V*_*GG1*_ and *V*_*GG2*_ are higher than the threshold voltage (*V*_*th*_), the whole channel region is conductive and the Schottky barrier is lowered because of the simultaneous up-shift of the graphene Fermi level, which results in n-type conductance of the device. When the *V*_*GG1*_ is higher than the threshold voltage and *V*_*GG2*_ is smaller, carrier transport through the ReS_2_ channel is blocked because of the depletion of the negatively gated ReS_2_ region, while the Schottky barrier is still small, resulting in carrier injection of the positively gated channel (middle of Fig. 5(a)). When both gate voltages are within the off-state, carrier transport is impossible due to depletion of the channel and higher contact barrier, resulting in a significantly low off-current level. Figure 5(b) shows the transfer curves (*I*_*DS*_ − *V*_*GG1*_) of the ReS_2_ device with graphene split gates when *V*_*GG1*_ is varying while *V*_*GG2*_ is fixed. The transfer curves are plotted on a semi-log scale in Fig. 5(c). In the device, hBN was used as an ultrathin and ultraflat dielectric in the device geometry, which is appropriate for “all-2D” devices. A small operating voltage (*V*_*BG*_ < 3 V) and low subthreshold swing (SS) of <300 mV/dec were obtained, which were an order of magnitude smaller in the ReS_2_ device on the 300 nm-thick SiO_2_ substrate which is calculated from the device described in Figs 2, 3. In the on-state (*V*_*GG1*_ > −1.5 V), conductance of the whole channel increases with increasing *V*_*GG1*_. However, in the off-state (*V*_*GG1*_ < −1.5 V), the off-current remains at a low level of 10^−12^ A at different *V*_*GG1*_ because the region where *V*_*GG1*_ is applied is completely off. Therefore, the on-current can be strongly modulated by *V*_*GG1*_ and *V*_*GG2*_. The on-off ratio modulation ranges from 10^2^ to 10^6^, which is beneficial for multifunctional applications (more clearly observed in the semi-log scale plot of Fig. 5(c)). Regardless of a gap (~200 nm), it is validated that the device operates reliably without significant deterioration of the performance, which have been also verified in previous reports^29,32^.

**Figure 6:**
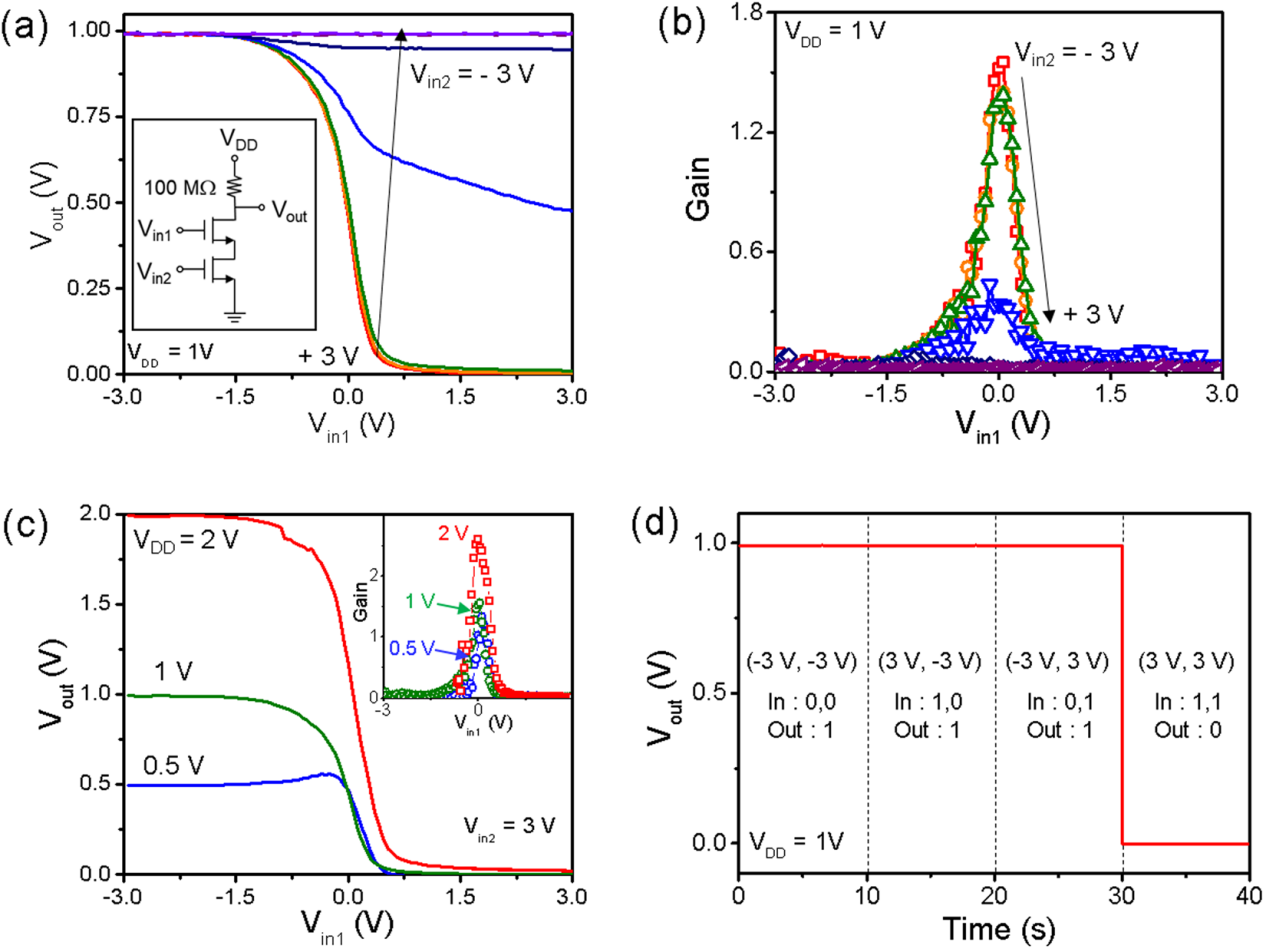
Logic device characteristics of ReS_2_ FET with graphene split gates. (**a**) Voltage transfer curves (VTCs) of GG1 region (*V*_*out*_ − *V*_*in1*_) at *V*_*DD*_ = 1 and varying *V*_*in2*_. Inset shows circuit diagram of logic device. External load is 100 MΩ. At *V*_*in2*_ > 1 V, VTC exhibits mirror symmetry. (**b**) Gain extracted by VTC as function of *V*_*in2*_. Maximum gain is 1.6 at *V*_*in2*_ = −3 V. (**c**) VTC at varying *V*_*DD*_ and fixed *V*_*in2*_ of 3 V. The inset shows gain extracted from VTC. (**d**) Time domain plot of output voltage for NAND logic gates.

The logic device characteristics of the ReS_2_ device with graphene split gates was measured, as shown in Fig. 6. As can be seen in the circuit diagram of Fig. 6(a), two graphene split gates are used as input signal gates. An external load of 100 MΩ, the middle off and on resistances value of the device, was used. Input terminals 1 (in1) and 2 (in2) were set as the GG1 and GG2, respectively. Because of high modulation ability, one half of the channel (controlled by one of split gates) operates as an independent inverter. The voltage transfer curves (VTCs) of Fig. 6(a) show the inverter characteristics. When *V*_*in1*_ varies with a fixed *V*_*in2*_, it can be clearly seen that the output signals are inverted, indicating that only half of the channel plays a role as an inverter. However, when the current is totally blocked by depletion of the half of the channel modulated by *V*_*in2*_, there is no inversion of the output signal. This means that the output signals can be effectively controlled by two input signals from the graphene split gates. The calculated gain values of the ReS_2_ inverter, defined as *dV*_*out*_*/dV*_*in*_, are shown in Fig. 6(b). Even though the gain value is relatively lower than those of previously reported 2D-materials-based inverters comprising two n-type and p-type transistors (similar to complementary metal oxide semiconductor (CMOS)), it is comparable to the gain values of unipolar-type inverters^5,27,35,36^. In addition, the supply voltage (*V*_*DD*_) used in this study is 1 V and the dielectric constant (ε_r_ = 3–4) of hBN is relatively small compared to previous studies^37,38^. Therefore, the gain can be further enhanced by increasing *V*_*DD*_ as shown in the inset in Fig. 6(c). In this study, *V*_*DD*_ was set as 1 V because the VTC exhibits a mirror reflection and a gain larger than 1 (unity gain), as shown in Fig. 6(c), compared to lower or higher *V*_*DD*_.

To utilize ReS_2_ devices as NAND logic devices, output voltages were measured under different combinations of input states, as shown in Fig. 6(d). Because the output voltage is linked to the *V*_*DD*_, a logic state of 1 can be realized when either *V*_*in1*_ or *V*_*in2*_ is −3 V (defined as an input logic state of 0). For *V*_*out*_ of state 0, positive input voltage of 3 V was applied to both graphene split gates of GG1 and GG2. If a gate voltage smaller than −1.5 V is applied to one of the two gates, the whole device is non-conducting. Therefore, ReS_2_ devices with graphene split gates can be used as a NAND gate, one of the universal logic gates. It should be noted that a NAND gate was fabricated on a single 2D channel material, which is distinct from other reports using two transistors regardless of the carrier type. Output voltages measured at different combinations of the two input signals are shown in Fig. 6(d). This indicates that a single channel ReS_2_ device with graphene split gates operates well as a NAND gate. Unlike bulk materials, gate tunability of 2D semiconducting materials enables independent control of the separated channel regions by split gates. Therefore, NAND logic device can be fabricated within single 2D channel, which is clearly different from conventional logic gates that require more than two separate transistors.

## Discussion

In conclusion, we fabricated high performance ReS_2_ transistors by using graphene and hBN as electrode and dielectric, respectively. The high field effect mobility of 35 cm^2^/V·s and high on-off ratio of 10^6^ were obtained by suppression of Fermi level pinning at the graphene-ReS_2_ junction and reduction of charged impurity scattering. For the “all-2D” integration, we also fabricated ReS_2_ devices with graphene split gates for logic devices. The graphene split gates and bottom hBN enables us to locally and effectively modulate channel regions. By independently changing gate voltages from the two split gates, the device conductance can be controlled with a higher degree of freedom: the whole device is turned off by applying a negative voltage to only one gate, while a positive gate voltage is applied to both split gates to turn-on the device. By utilizing local tunability of the channel by split gates, it was shown that the ReS_2_ device with graphene split gates can be used as an efficient NAND gate, which is one of the most essential elements in integrated circuits. The vdW heterostructure NAND gate comprising a single transistor fabricated in this work provides a further step toward realization of “all-2D” circuitry for flexible and transparent electronic applications.

## Methods

### Device fabrication

PDMS transfer First, the PDMS base (Sylgard 184) was cured with 10 wt% of curing agent at 120 °C for 2 h. We then mechanically exfoliated hBN, which is the bottom of the heterostructure on SiO_2_/Si chips, and exfoliated other 2D materials, such as graphene and ReS_2_, separately onto each cured PDMS flake on glass slides. After careful alignment with a micromanipulator in the transfer stage, the PDMS with 2D materials were attached and detached, as carefully and slowly as possible, at room temperature.

### E-beam lithography

To pattern metal electrodes on the as-stacked heterostructure or gap on the graphene for the split gate, we performed e-beam lithography with a Raith Pioneer 2 nanolithography system.

### Metal deposition

We deposited metal electrodes (Cr 1 nm/Au 50 nm or Cr 1 nm/ Pd 20 nm/Au 30 nm) with an e-beam evaporator (Korea Vacuum Tech.) in a high vacuum, of approximately 10^−8^ Torr, to create the interconnection to the pads for the electrical measurement from the graphene electrodes, where 1 nm of Cr is an adhesive layer. For the lift-off process, the samples were soaked in acetone overnight.

### Reactive ion etching

We etched the developed region after the e-beam lithography using O_2_ RIE (Femto Science) for only 5 s to prevent etching of e-beam resist. (100 kHz, 100 W in 10^−2^ Torr of O_2_) We then put the samples in acetone to remove the e-beam resist.

### Electrical characterization

All electrical measurements were carried out by semiconductor parameter analyzers at room temperature under ambient condition. (Keithely 4200, Keithely for FET characterization in Figs 3, 5; and HP 4155 C, Agilent Technologies for logic device measurement in Fig. 6)

## Supplementary information

Supplementary information for “All-2D ReS<Subscript>2</Subscript> transistors with split gates for logic circuitry”

## Data Availability

The authors declare that all data supporting the findings of this study are available within the paper and its supplementary information files.

## References

[1] Radisavljevic, B., Radenovic, A., Brivio, J., Giacometti, V. & Kis, A. Single-layer MoS_2_ transistors. *Nat. Nanotechnol.* **6**, 147 (2011). 21278752 10.1038/nnano.2010.279

[2] Lopez-Sanchez, O., Lembke, D., Kayci, M., Radenovic, A. & Kis, A. Ultrasensitive photodetectors based on monolayer MoS_2_. *Nat. Nanotechnol.* **8**, 497 (2013). 23748194 10.1038/nnano.2013.100

[3] Cheng, R. *et al*. Electroluminescence and photocurrent generation from atomically sharp WSe_2_/MoS_2_ heterojunction p–n diodes. *Nano Lett.* **14**(10), 5590–5597 (2014). 25157588 10.1021/nl502075n PMC4189621

[4] Tsai, M.-L. *et al*. Monolayer MoS_2_ heterojunction solar cells. *ACS Nano* **8**(8), 8317–8322 (2014). 25046764 10.1021/nn502776h

[5] Wang, H. *et al*. Integrated circuits based on bilayer MoS_2_ Transistors. *Nano Lett.* **12**(9), 4674–4680 (2012). 22862813 10.1021/nl302015v

[6] Desai, S. B. *et al*. MoS_2_ transistors with 1-nanometer gate lengths. *Science* **354**(6308), 99–102 (2016). 27846499 10.1126/science.aah4698

[7] Xu, K. *et al*. Sub-10 nm nanopattern architecture for 2D material field-effect transistors. *Nano Lett.* **17**(2), 1065–1070 (2017). 28092953 10.1021/acs.nanolett.6b04576

[8] Liu, Y. *et al*. Van der Waals heterostructures and devices. *Nat. Rev. Mater.* **1**, 16042 (2016).

[9] Wang, L. *et al*. Shepard, K. L.; Dean, C. R., One-dimensional electrical contact to a two-dimensional material. *Science* **342**(6158), 614–617 (2013). 24179223 10.1126/science.1244358

[10] Lee, C.-H. *et al*. Atomically thin p–n junctions with van der Waals heterointerfaces. *Nat. Nanotechnol.* **9**, 676 (2014). 25108809 10.1038/nnano.2014.150

[11] Son, J. *et al*. Atomically precise graphene etch stops for three dimensional integrated systems from two dimensional material heterostructures. *Nat. Commun.* **9**(1), 3988 (2018). 30266948 10.1038/s41467-018-06524-3 PMC6162276

[12] Splendiani, A. *et al*. Emerging photoluminescence in monolayer MoS_2_. *Nano Lett.* **10**(4), 1271–1275 (2010). 20229981 10.1021/nl903868w

[13] Tongay, S. *et al*. Monolayer behaviour in bulk ReS_2_ due to electronic and vibrational decoupling. *Nat. Commun.* **5**, 3252 (2014). 24500082 10.1038/ncomms4252

[14] Zhang, E. *et al*. ReS_2_-based field-effect transistors and photodetectors. *Adv. Func. Mater.* **25**(26), 4076–4082 (2015).

[15] Park, J. Y. *et al*. Contact effect of ReS_2_/metal interface. *ACS Appl. Mater. Inter.* **9**(31), 26325–26332 (2017). 10.1021/acsami.7b06432 28718280

[16] Liu, F. *et al*. Highly sensitive detection of polarized light using anisotropic 2D ReS_2_. *Adv. Func. Mater.* **26**(8), 1169–1177 (2016).

[17] Shim, J. *et al*. High-Performance 2D Rhenium Disulfide (ReS_2_) Transistors and photodetectors by oxygen plasma treatment. *Adv. Mater.* **28**(32), 6985–6992 (2016). 27206245 10.1002/adma.201601002

[18] Xu, K. *et al*. Sulfur vacancy activated field effect transistors based on ReS_2_ nanosheets. *Nanoscale* **7**(38), 15757–15762 (2015). 26352273 10.1039/c5nr04625d

[19] Dathbun, A. *et al*. Large-area CVD-grown sub-2 V ReS_2_ transistors and logic gates. *Nano Lett.* **17**(5), 2999–3005 (2017). 28414455 10.1021/acs.nanolett.7b00315

[20] Kim, C. *et al*. Fermi level pinning at electrical metal contacts of monolayer molybdenum dichalcogenides. *ACS Nano* **11**(2), 1588–1596 (2017). 28088846 10.1021/acsnano.6b07159

[21] Kwon, J. *et al*. Thickness-dependent Schottky barrier height of MoS_2_ field-effect transistors. *Nanoscale* **9**(18), 6151–6157 (2017). 28447707 10.1039/c7nr01501a

[22] Cui, X. *et al*. Multi-terminal transport measurements of MoS_2_ using a van der Waals heterostructure device platform. *Nat. Nanotechnol.* **10**, 534 (2015). 25915194 10.1038/nnano.2015.70

[23] Liu, Y., Stradins, P. & Wei, S.-H. Van der Waals metal-semiconductor junction: Weak Fermi level pinning enables effective tuning of Schottky barrier. *Sci. Adv*. **2** (4) (2016). 10.1126/sciadv.1600069 PMC4846439 27152360

[24] Dean, C. R. *et al*. Boron nitride substrates for high-quality graphene electronics. *Nat. Nanotechnol.* **5**, 722 (2010). 20729834 10.1038/nnano.2010.172

[25] Liu, X. *et al*. P-type polar transition of chemically doped multilayer MoS_2_ transistor. *Adv. Mater.* **28**(12), 2345–2351 (2016). 26808483 10.1002/adma.201505154

[26] Jeon, P. J. *et al*. Low power consumption complementary inverters with n-MoS_2_ and p-WSe_2_ dichalcogenide nanosheets on glass for logic and light-emitting diode circuits. *ACS Appl. Mater. Inter.* **7**(40), 22333–22340 (2015). 10.1021/acsami.5b06027 26399664

[27] Liu, E. *et al*. Integrated digital inverters based on two-dimensional anisotropic ReS_2_ field-effect transistors. *Nat. Commun.* **6**, 6991 (2015). 25947630 10.1038/ncomms7991 PMC4432591

[28] Cho, A.-J., Park, K. C. & Kwon, J.-Y. A high-performance complementary inverter based on transition metal dichalcogenide field-effect transistors. *Nanoscale Res. Lett.* **10**(1), 115 (2015). 25852410 10.1186/s11671-015-0827-1 PMC4385225

[29] Ross, J. S. *et al*. Electrically tunable excitonic light-emitting diodes based on monolayer WSe_2_ p–n junctions. *Nat. Nanotechnol.* **9**, 268 (2014). 24608230 10.1038/nnano.2014.26

[30] Baugher, B. W. H., Churchill, H. O. H., Yang, Y. & Jarillo-Herrero, P. Optoelectronic devices based on electrically tunable p–n diodes in a monolayer dichalcogenide. *Nat. Nanotechnol.* **9**, 262 (2014). 24608231 10.1038/nnano.2014.25

[31] Lemme, M. C. *et al*. Gate-activated photoresponse in a graphene p–n junction. *Nano Lett.* **11**(10), 4134–4137 (2011). 21879753 10.1021/nl2019068

[32] Pospischil, A., Furchi, M. M. & Mueller, T. Solar-energy conversion and light emission in an atomic monolayer p–n diode. *Nat. Nanotech.* **9**, 257 (2014). 10.1038/nnano.2014.14 24608229

[33] Groenendijk, D. J. *et al*. Photovoltaic and photothermoelectric effect in a double-gated WSe_2_ Device. *Nano Lett.* **14**(10), 5846–5852 (2014). 25232893 10.1021/nl502741k

[34] Kim, I. S. *et al*. Influence of stoichiometry on the optical and electrical properties of chemical vapor deposition derived MoS_2_. *ACS Nano* **8**(10), 10551–10558 (2014). 25223821 10.1021/nn503988x PMC4212723

[35] Tosun, M. *et al*. High-gain inverters based on WSe_2_ complementary field-effect transistors. *ACS Nano* **8**(5), 4948–4953 (2014). 24684575 10.1021/nn5009929

[36] Cheng, R. *et al*. Few-layer molybdenum disulfide transistors and circuits for high-speed flexible electronics. *Nat. Commun.* **5**, 5143 (2014). 25295573 10.1038/ncomms6143 PMC4249646

[37] Kim, K. K. *et al*. Synthesis and characterization of hexagonal boron nitride film as a dielectric layer for graphene devices. *ACS Nano* **6**(10), 8583–8590 (2012). 22970651 10.1021/nn301675f

[38] Laturia, A., Van de Put, M. L. & Vandenberghe, W. G. Dielectric properties of hexagonal boron nitride and transition metal dichalcogenides: from monolayer to bulk. *npj 2D Mater. Appl.* **2**(1), 6 (2018).

