# Correction: All-2D ReS_2_ transistors with split gates for logic circuitry

**DOI:** 10.1038/s41598-025-05614-9

**Published:** 2025-06-26

**Authors:** Junyoung Kwon, Yongjoon Shin, Hyeokjae Kwon, Jae Yoon Lee, Hyunik Park, Kenji Watanabe, Takashi Taniguchi, Jihyun Kim, Chul-Ho Lee, Seongil Im, Gwan-Hyoung Lee

**Affiliations:** 1 Department of Materials Science and Engineering, Yonsei University, Seoul, 03722 Korea; 2 Department of Materials Science and Engineering, Seoul National University, Seoul, 08826 Korea; 3 vdWMRC, Department of Physics, Yonsei University, Seoul, 03722 Korea; 4 KU‐KIST Graduate School of Converging Science and Technology, Korea University, Seoul, 02841 Korea; 5 Department of Chemical and Biological Engineering, Korea University, Seoul, 02841 Korea; 6 National Institute for Materials Science, Ibaraki, 305-0044 Japan

Correction to: *Scientific Reports* 10.1038/s41598-019-46730-7 Published Online 17 July 2019.

The original version of this Article contains an error in the name of the author Yongjoon Shin, which was incorrectly given as Yongjun Shin.

