# Supplementary material for: All-2D ReS2 transistors with split gates for logic circuitry
Source: Sci Rep. 2019 Jul 17;9:10354. doi: 10.1038/s41598-019-46730-7 (PMC6637167; doi:10.1038/s41598-019-46730-7)
Supplement: Supplementary file 1 — Supplementary information for “All-2D ReS2 transistors with split gates for logic circuitry” [file 41598_2019_46730_MOESM1_ESM.pdf]

Supplementary Information for

All-2D ReS_2 transistors with split gates for logic circuitry

Junyoung Kwon,[a] *Yongjun Shin,*[b] *Hyeokjae Kwon,*[c] *Jae Yoon Lee,*[d] *Hyunik Park,*[e] *Kenji Watanabe,*[f] *Takashi Taniguchi,*[f] *Jihyun Kim,*[e] *Chul-Ho Lee,*[d] *Seongil Im*[c] *and Gwan-Hyoung Lee**[,b]

a. Department of Materials Science and Engineering, Yonsei University, Seoul 03722, Korea

b. Department of Materials Science and Engineering, Seoul National University, Seoul 08826, Korea

c. vdWMRC, Department of Physics, Yonsei University, Seoul 03722, Korea

d. KU-KIST Graduate School of Converging Science and Technology, Korea University, Seoul 02841, Korea

e. Department of Chemical and Biological Engineering, Korea University, Seoul 02841, Korea

f. National Institute for Materials Science, Ibaraki 305-0044, Japan

*Corresponding e-mail : gwanlee@snu.ac.kr

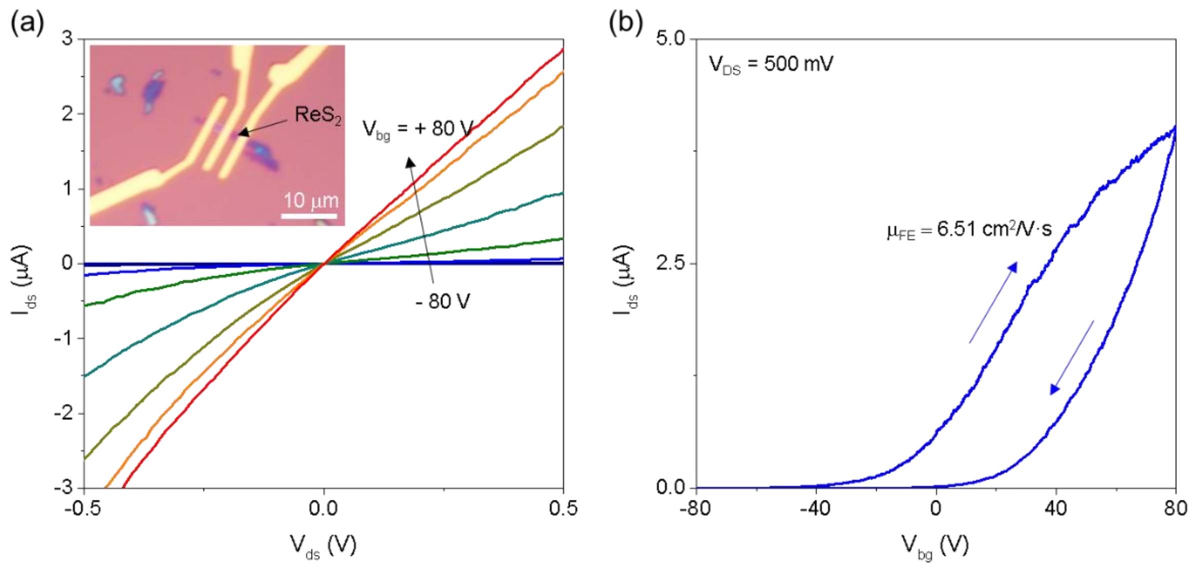

Fig. S1. Comparison of the performance with the same ReS_2 FET directly contacted by metal (Cr 1nm / Au 50 nm) on standard SiO_2 (285 nm) / Si substrate, utilizing the same process as the all-2D ReS_2 FET. (a) Output curves of the reference device at various back gate voltages. The S-shaped curves at low carrier concentration indicate that the contact is not Ohmic, while the same device with graphene electrodes show perfectly linear output curves. (b) Transfer curves measured in both forward and reverse direction of gate voltage. Relatively low field effect mobility of 6.51 $cm^2/V \cdot s$ for electron is obtained due to both contact effect and charged impurities from the substrate.

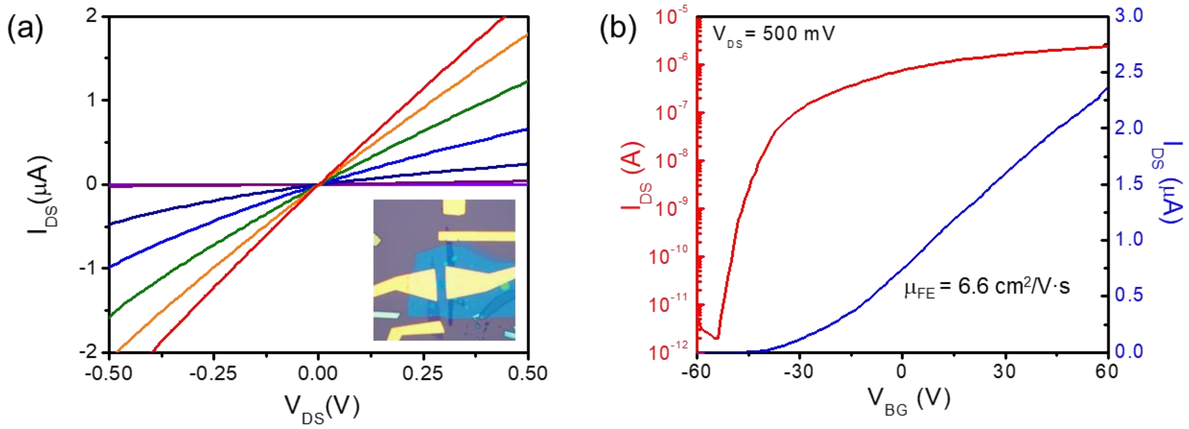

Fig. S2. Electrical measurement of $ReSe_2$ FET on hBN with graphene electrodes. (a) Output curves (I_{DS} $-V_{BG}$) of a $ReSe_2$ FET. The linear output curves indicate that graphene-$ReSe_2$ junction forms Ohmic contact. The inset shows the optical microscope image of the fabricated $ReSe_2$ FET. (b) Transfer curves ($I_{DS} - V_{BG}$) of the $ReSe_2$ FET in linear scale (black) and semi-log scale (red) with V_{DS} = 500 mV. The device shows high field-effect mobility of 6.6 cm^2 /V·s and high on-off ratio of 10^6. Field effect mobility of $ReSe_2$ is lower than that of ReS_2 with the same device geometry.

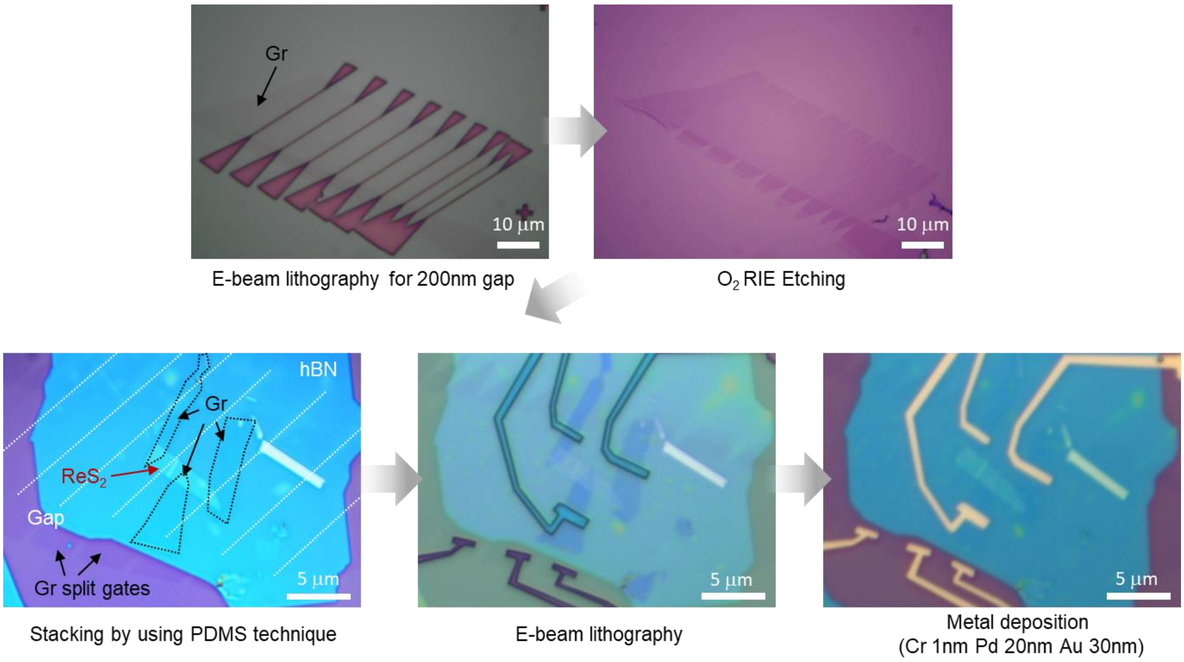

Fig. S3. Fabrication process of ReS_2 FET with graphene split gates. After mechanical exfoliation of graphene on SiO_2 substrate, the graphene was patterned into several cuts with nanogap of 200 nm by e-beam lithography. To cut the graphene electrode, oxygen plasma RIE was used, followed by removal of e-beam resist (PMMA) with acetone. Then, the flakes of hBN, ReS_2, graphene were sequentially transferred onto the gapped graphene electrodes by using PDMS stamp technique described in Fig. 1. For interconnects to graphene electrodes, metal electrodes were patterned by e-beam lithography, followed by deposition of Cr 1 nm, Pd 20 nm, and Au 30 nm using e-beam evaporator.
